# Optimization of TaqMan-based quantitative PCR diagnosis for *Entamoeba histolytica* using droplet digital PCR

**DOI:** 10.1371/journal.pntd.0012935

**Published:** 2025-06-05

**Authors:** Akira Kawashima, Yasuaki Yanagawa, Takayuki Chikata, Rieko Shimogawara, Daisuke Mizushima, Kiyoto Tsuchiya, Kenji Yagita, Hiroyuki Gatanaga, Koji Watanabe

**Affiliations:** 1 AIDS Clinical Center, National Center for Global Health and Medicine, Japan Institute for Health Security, Tokyo, Japan; 2 The Joint Research Center for Human Retrovirus Infection Kumamoto University Campus, Kumamoto, Japan; 3 Department of Parasitology, National Institute of Infectious Diseases, Japan Institute for Health Security, Tokyo, Japan; 4 Department of Microbiology and Immunology, Stanford University School of Medicine, Stanford, California, United States of America; 5 Division of Host Defense Mechanism, Tokai University School of Medicine, Kanagawa, Japan; University of California San Diego, UNITED STATES OF AMERICA

## Abstract

**Background:**

TaqMan-probed quantitative PCR (qPCR) is highly valued for diagnosing *Entamoeba histolytica* infections (amebiasis). However, unclear cycle threshold (Ct) values often yield low-titer positive results, complicating interpretation. This study aimed to optimize qPCR primer-probe sets with logically determined cut-off Ct value using droplet digital PCR (ddPCR).

**Methodology/Principal Findings:**

Amplification efficacy was evaluated using ddPCR by measuring absolute positive droplet counts (APD) and mean fluorescence intensity at different PCR cycles and annealing temperatures (AT). A primer-probe specific cut-off Ct value was determined from a standard curve by correlating Ct values with APD. Twenty primer-probe sets targeting small subunit rRNA gene regions (X64142) were designed from previous papers. Amplification efficacy remained consistent at high PCR cycles (50 cycles), but differed at lower PCR cycles (30 cycles), identifying five sets with higher amplification efficiency than other candidates. Of these, only two sets maintained efficiency at higher AT (62°C). Ct value was inversely proportional to the square of APD, defining the specific cut-off Ct value as 36 cycles. Selected primer-probe set with a cut-off effectively differentiated *E. histolytica* infection in clinical specimens. However, discordant results between Ct value and APD were seen in some cases with high Ct value. Shotgun metagenomic sequencing suggested microbial-independent false positive reactions contributed to these discrepancies, although specific reactants were unidentified.

**Conclusions/Significance:**

The combination use of ddPCR with qPCR revealed that false positive reactions of qPCR and/or ddPCR commonly happen in stool specimens. Also, this study emphasizes the value of ddPCR for establishing accurate cut-off values with efficient primer-probes.

## Introduction

Amebiasis, transmitted orally, leads to diseases such as colitis and liver abscess [1]. In developing countries, transmission typically occurs via the ingestion of food and water contaminated with feces, which is the second leading cause of parasite-related death worldwide [2]. More recently, it has been reported that amebiasis is spreading throughout Asian and European developed countries as a sexually transmitted infection [1,3–8].

Among the currently available laboratory tests for *Entamoeba histolytica,* TaqMan-probed quantitative PCR (qPCR) is commonly used for clinical diagnosis and epidemiological studies because of its high sensitivity [9]. However, the specificity of qPCR is still in doubt, especially when it is used for stool samples. We often face unexpected positive results with high Ct values in clinical settings, such as unexpected multiple pathogens detected by the intestinal panel of multiplex stool PCR system [10], and unexpectedly high frequency of asymptomatic carriers in epidemiological research [11–13]. Previously, a high Ct was considered a feature of asymptomatic carriers rather than symptomatic diseases; however, recent studies identified the clinical severity of intestinal infection is not necessarily correlated with the Ct value [14,15]. Taken together, the efficacy of qPCR should be assessed precisely, and cut-off Ct values should be set by logical strategy for better clinical practice.

Droplet digital PCR (ddPCR) is a third-generation PCR method that provides absolute quantification, enabling the partitioning of a sample into over 10,000 droplets, with each droplet serving as an independent reaction [14,15]. The advantage of ddPCR is that fluorescence intensity is measured for each droplet, whereas qPCR measures the sum of the total template DNA in each sample [16,17]. The fluorescence of each droplet represents an amplificon from one template DNA. The utility of ddPCR has been proven for clinical samples [16], although its use is still limited in the laboratory because of its cost and slightly complicated operation. In a study comparing ddPCR and qPCR, ddPCR accurately quantified samples, even at low concentrations, and it was less affected by contamination than qPCR [18,19]. However, its utility has not been fully assessed in previous studies [17,20].

The aim of this study was to optimize the diagnostic value of qPCR. For this, we newly applied ddPCR for evaluating the amplification efficacy of primer-probe sets, and determining cut-off Ct values by the logical approach.

## Methods

### Ethics statement

This study was conducted following ethical approval from the Institutional Review Board of the National Center for Global Health and Medicine (NCGM-S-004658). Written informed consent was obtained from all participants. A total of 66 clinical samples (60 stool samples, 3 intestinal fluids, 2 pleural effusions and 1 liver abscess) were collected from patients with suspected *Entamoeba histolytica* infections (see detailed information in S1 Data). After collecting clinical samples, the specimens were immediately processed for DNA extraction to minimize DNA degradation.

### Reference strain of *E. histolytica* for standard

For optimization of PCR assays, a laboratory strain of *E. histolytica* (HM1:IMSS clone 6) was used. HM1:IMSS clone 6 was generated from the original strain of HM1:IMSS in our laboratory, which has been maintained in axenic YIMDHA-S medium for more than 10 years [21]. We collected 2 x 10^7^ log-phase growing trophozoites in PBS. Thereafter, DNA extraction was performed using QIAamp DNA Kits for DNA Extraction (Qiagen, Hilden, Germany) according to the manufacture’s instruction. Finally, the DNA template was eluted in 200 μL of DNase/RNase-free water, which contains *E. histolytica* derived DNA of 100,000 trophozoites/μL as a stock template solution. The stocked template solution was diluted prior to the experiments, and used for the standards for qPCR, and positive control for ddPCR and conventional PCR.

### DNA extraction

DNA was extracted from clinical specimens using a QIAamp Fast DNA Stool Mini Kit (Qiagen, Hilden, Germany) according to the manufacturer’s protocol, which includes an inhibitor removal step optimized for PCR analysis. On the final step for elution, template DNA was prepared in 50 μL of DNase/RNase free water. Prior to PCR for *E. histolytica*, amplification efficacy was assessed by qPCR reaction of an internal positive control (Nippon Gene, Toyama, Japan) to confirm that PCR inhibitory factors were not contaminated in the template solution. The extracted DNA was stored at −20°C until analysis.

### Primer and probe selection

We selected forward and reverse primers as well as probes from the previous papers, whose diagnostic utility was confirmed using clinical specimen. All primer-probe sets target the small subunit rRNA gene (X64142). From them, we designed primer-probe sets (Tables 1 and 2) [9,22–25].

**Table 1:** Sequences of primers used in this study.

A.Sequences of forward (5’) primers		
Name	Sequences	Reference
Forward A (ForA)	GCGGACGGCTCATTATAACA	[9,23]
Forward B (ForB)	CAGTAATAGTTTCTTTGGTTAGTAAAA	[22]
Forward C (ForC)	AAATGGCCAATTCATTCAATGA	[24]
B.Sequences of reverse (3’) primers		
Name	Sequences	Reference
Reverse A (RevA)	GTCCTCGATACTACCAAC	[9]
Reverse B (RevB)	CTTAGAATGTCATTTCTCAATTCAT	[22]
Reverse C (RevC)	ATTGTCGTGGCATCCTAACTCA	[23]
Reverse D (RevD)	CATTGGTTACTTGTTAAACACTGTGTG	[24]
C.Sequences of TaqMan probes		
Name	Sequence	Reference
Probe A (ProA)	GAATGAATTGGCCATTT	[9,23]
Probe B (ProB)	GTTTGTATTAGTACAAAATGGC	[22]
Probe C (ProC)	AGGATGCCACGACAA	[24]

**Table 2:** Primer-probe sets used in the present study.

Primer-probe set	5’ Primer*	3’ Primer**	Amplicon length	Probe***
Set 1	ForA	RevB	151 bp	ProB
Set 2	ForA	RevC	173 bp	ProB
Set 3	ForA	RevC	173 bp	ProA
Set 4	ForA	RevD	207 bp	ProB
Set 5	ForA	RevD	207 bp	ProA
Set 6	ForA	RevD	207 bp	ProC
Set 7	ForA	RevA	247 bp	ProB
Set 8	ForA	RevA	247 bp	ProA
Set 9	ForA	RevA	247 bp	ProC
Set 10	ForB	RevB	135 bp	ProB
Set 11	ForB	RevC	157 bp	ProB
Set 12	ForB	RevC	157 bp	ProA
Set 13	ForB	RevD	191 bp	ProB
Set 14	ForB	RevD	191 bp	ProA
Set 15	ForB	RevD	191 bp	ProC
Set 16	ForB	RevA	231 bp	ProB
Set 17	ForB	RevA	231 bp	ProA
Set 18	ForB	RevA	231 bp	ProC
Set 19	ForC	RevD	99 bp	ProC
Set 20	ForC	RevA	139 bp	ProC

*The 5’ primer was selected from Table 1 A. ** The 3’ primer was selected from Table 1 B. ***The probe was selected from Table 1 C.

### ddPCR protocol

Each reaction consisted of 10 μL ddPCR Supermix for Probes (No dUTP; Bio-Rad, Munich, Germany), 18 pmol of each primer, 5 pmol of probes, and 1 μL DNA template, which are the optimum concentrations specified in the package insert of PCR enzyme. Also, we confirmed the stable amplification efficacy in all experiments using positive control (HM1:IMSS clone 6). The total reaction volume was adjusted to 20 μL.

Droplets were generated with a QX200 Droplet Generator, transferred to a 96-well PCR plate, and amplified on a C1000 Touch Thermal Cycler under conditions of initial denaturation at 95°C for 10 minutes, followed by 20–50 cycles at 94°C for 30 seconds, 59–62°C for 1 minute, and a final extension at 98°C for 10 minutes. The PCR cycle was set at 50 cycles for clinical samples, whereas various cycle numbers were examined for primer-probe set optimization. Annealing temperatures of 59°C and 62°C were selected after an initial optimization across 55–65°C. All reactions included no-template controls. Each sample was run in quadruplicate for reproducibility.

Amplified droplets were analyzed using a QX200 Droplet Reader to quantify absolute positive droplet counts (APD) and mean fluorescence intensity (MFI) using a Bio-Rad QX200 system.

### qPCR protocol

Each 20 μL reaction contained 10 μL TaqMan Fast Advanced Master Mix 2 × buffer (Thermo Fisher Scientific, Waltham, MA, USA), 5 pmol of each primer and probe, 2 μL template DNA. These concentrations follow the manufacturer’s protocol. Thermal cycling included an initial denaturation at 95°C for 30 seconds, followed by 40 cycles at 95°C for 5 seconds, and 59–62°C for 30 seconds. A temperature range was evaluated during primer-probe set optimization. Each sample was run in duplicate, and a standard curve was generated by the serial dilution of template DNA from the reference standard (HM1:IMSS clone 6) for each experiment. Ct values were analyzed using QuantStudio Design & Analysis software.

### Shotgun metagenome sequencing analysis for discordant PCR cases

Shotgun metagenome sequencing was performed on discordant PCR results using the DNBSEQ platform. Three discordant PCR cases were selected for shotgun metagenomic sequencing based on sufficient DNA yield and representativeness of discrepant patterns observed between qPCR and ddPCR. DNA libraries were prepared using a KAPA HyperPlus Kit according to the manufacturer’s protocol, with additional purification for high-quality DNA. Libraries were quantified with BioAnalyzer 2100 (Agilent Technologies) and sequenced as paired-end reads (2 × 150 bp). Sequencing data were processed using a bioinformatics pipeline. Raw reads were subjected to adapter trimming, removal of low-quality sequences (limit = 0.05), and exclusion of reads shorter than 105 bp. Filtered reads were mapped to the *E. histolytica* reference sequences, including the small subunit rRNA gene (X64142) and HM-1:IMSS genome (JCVI-ESG2-1.0) using CLC Genomics Workbench with a length and similarity fraction threshold ≥ 0.8. Reads containing primer-homologous regions (allowing up to two mismatches) were identified and assembled *de novo* into contigs. Contigs were analyzed using BLAST against the NCBI core nt database to determine taxonomic origins based on significant alignments (E-values <10^5^).

### Statistical analysis

Statistical analyses were performed using GraphPad Prism (v10.2.3). The Pearson correlation coefficient was calculated to assess the relationship between APD counts and Ct values, with statistical significance defined as p < 0.05. Discrepancies were compared by one-way ANOVA followed by compact letter display for amplification efficiencies.

## Results

### Designing primer-probe sets for clinical specimens

For the clinical diagnosis of *E. histolytica,* primer-probe sets targeting the small subunit rRNA gene (X64142) were designed because of its high sensitivity. We designed twenty primer-probe sets in combination with primers and probes, all of which were proven for clinical utility in previous papers (Tables 1 and 2) [9,22–25]. Also, these primer-probe sets met the following criteria: 1. The size of the amplicon is < 300 bp for primers, and 2. Double quencher with ZEN enhancement can be designed for the probe.

### Comparison of amplification efficacy of each primer-probe set by ddPCR

#### Determination of highly sensitive primer-probe sets.

**Fig 1:**
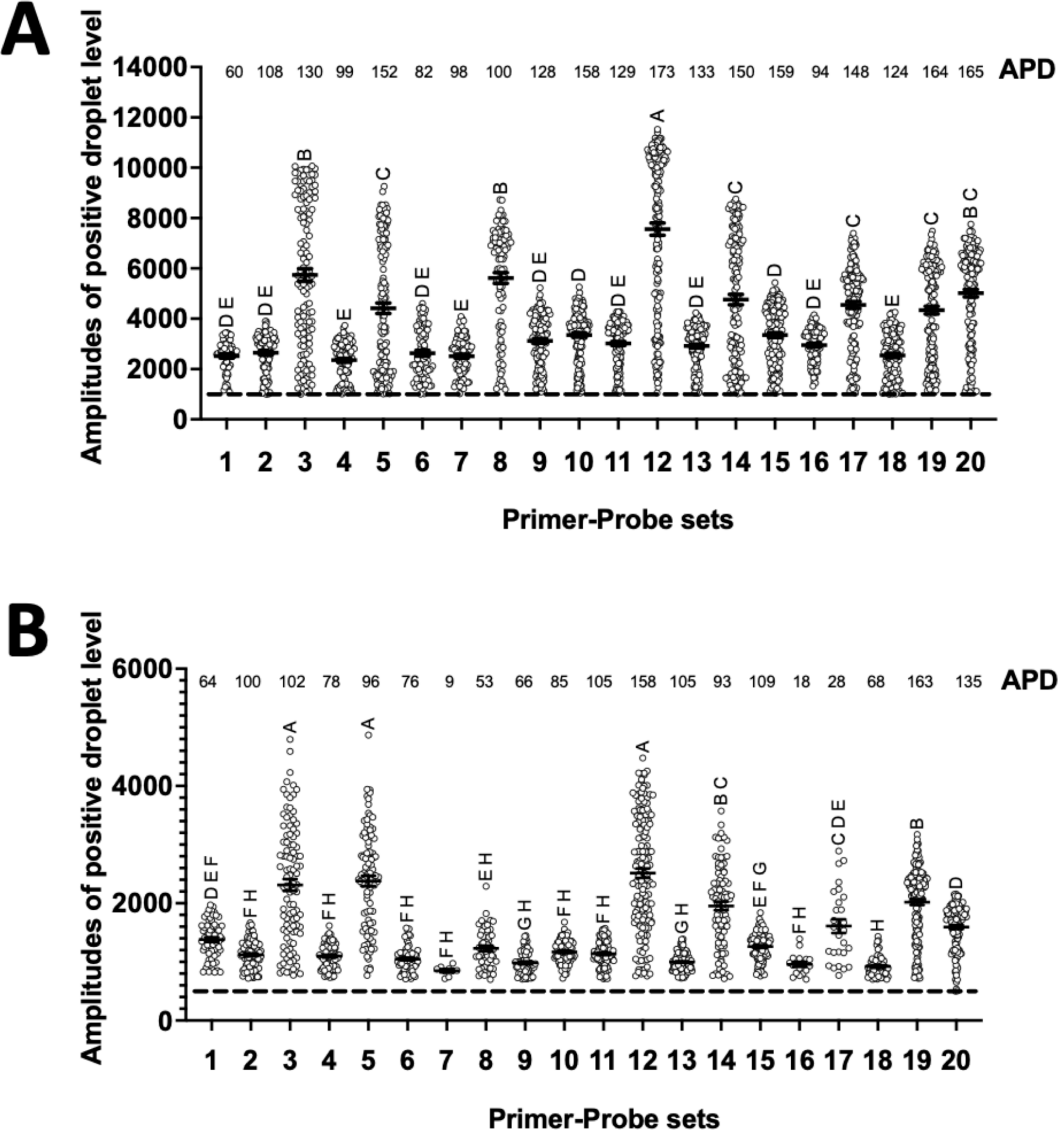
Evaluation of the amplification efficacy of primer-probe sets by droplet digital PCR (ddPCR). Absolute positive droplet counts (APD) and mean fluorescent intensity (MFI) were assessed by droplet digital PCR (ddPCR), which were compared among 20 prime-probe sets targeting the same *E. histolytica* gene (X64142). Assays were performed using different PCR cycles: 50 cycles (A) and 30 cycles (B). All conditions other than PCR cycles were the same for all experiments. MFIs were compared by one-way ANOVA and presented as compact letter display (CPD) using GraphPad Prism software.

First, we performed ddPCR to compare the amplification efficacy of each primer-probe set. We fixed the annealing temperature (59°C) and template concentration (1000 trophozoites per microliter) for the experiment. The 1,000 trophozoites/μL DNA template was prepared from HM1:IMSS clone 6 (detailed description in the Methods section). On the other hand, PCR cycles were decreased by 10 cycles from the standard number of PCR cycles (50 cycles). With 50 cycles, APD counts were similarly high in all primer-probe sets, moreover, a comparison of the MFI of positive droplets was difficult (Fig 1A). Interestingly, as PCR cycles decreased, the differentiation of MFI became easier, although the MFI of all droplets decreased. When the cycle number was reduced to 30, APD counts were decreased in some primer-probe sets with relatively low MFI. Surprisingly, only five primer-probe sets (sets 3, 5, 12, 14, and 19) among 20 candidates maintained amplification efficacy, whose MFI levels were represented as “A” or “B” by the multiple comparison using GraphPad Prism software (Fig 1B).

#### Determination of primer-probe sets with high specificity.

**Fig 2:**
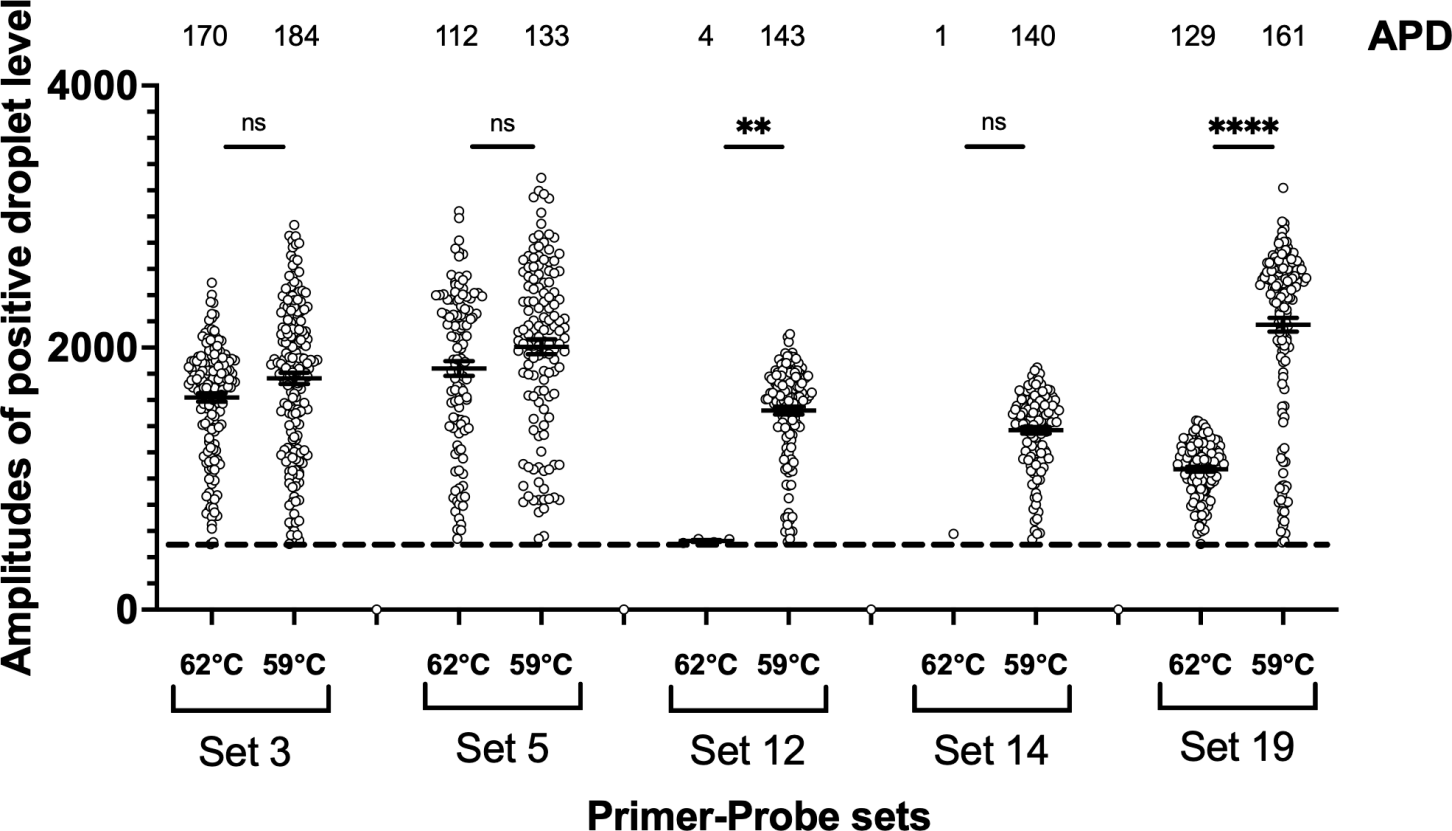
Evaluation of the amplification efficacy of five selected primer-probe sets at different annealing temperatures: 59°C versus 62°C.

**Fig 3:**
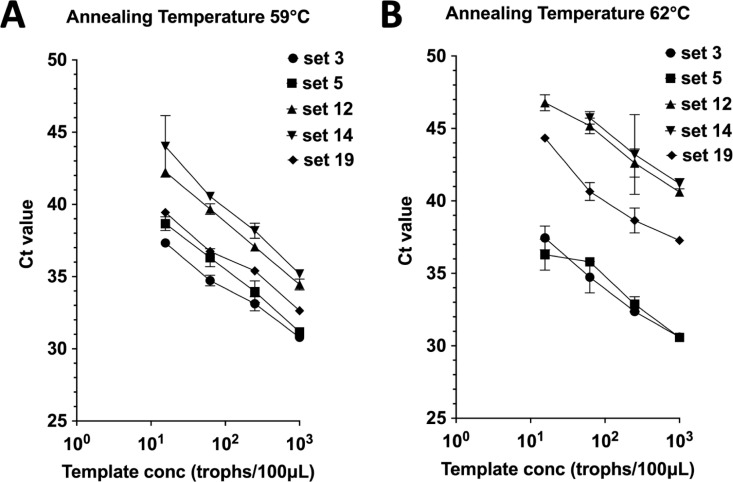
Evaluation of the diagnostic efficacy of TaqMan-probed quantitative PCR (qPCR). Ct values of five primer-probe sets were compared by the serial dilution of *E. histolytica* DNA. PCR was performed with 40 cycles at different annealing temperatures: 59°C (A), or 62°C (B).

Next, to identify primer-probe sets with low non-specific amplification in PCR, we compared the amplification efficacy at two different annealing temperatures, 59°C and 62°C (Fig 2). Amplification efficacy was not decreased in sets 3 and 5, whereas that of the other sets was significantly decreased with a higher annealing temperature. Finally, to assess our ddPCR strategy for choosing effective primer-probe sets, we performed qPCR assay using these candidate primer-probe sets (Fig 3A and 3B). As expected, sets 3 and 5 had lower Ct values compared with the other primer-probe sets, especially at higher annealing temperature. Sets 3 and 5 contain the same forward primer and probe, whereas the reverse primer is different (Table 2). In the precious report, it was shown that set 5 might amplify non-pathogenic *Entamoeba dispar,* possibly present in Japan, and the potentially pathogenic *E. moshkovskii* [9]. In the same paper, it was also confirmed that set 3 amplified *E. dispar*, and the different non-pathogenic *Entamoeba* species, *E. bangladeshii*. Therefore, we selected set 5 for further evaluation in the present study with consideration for future clinical use in Japan.

#### Determination of Cut-off Ct Values for TaqMan qPCR.

**Fig 4:**
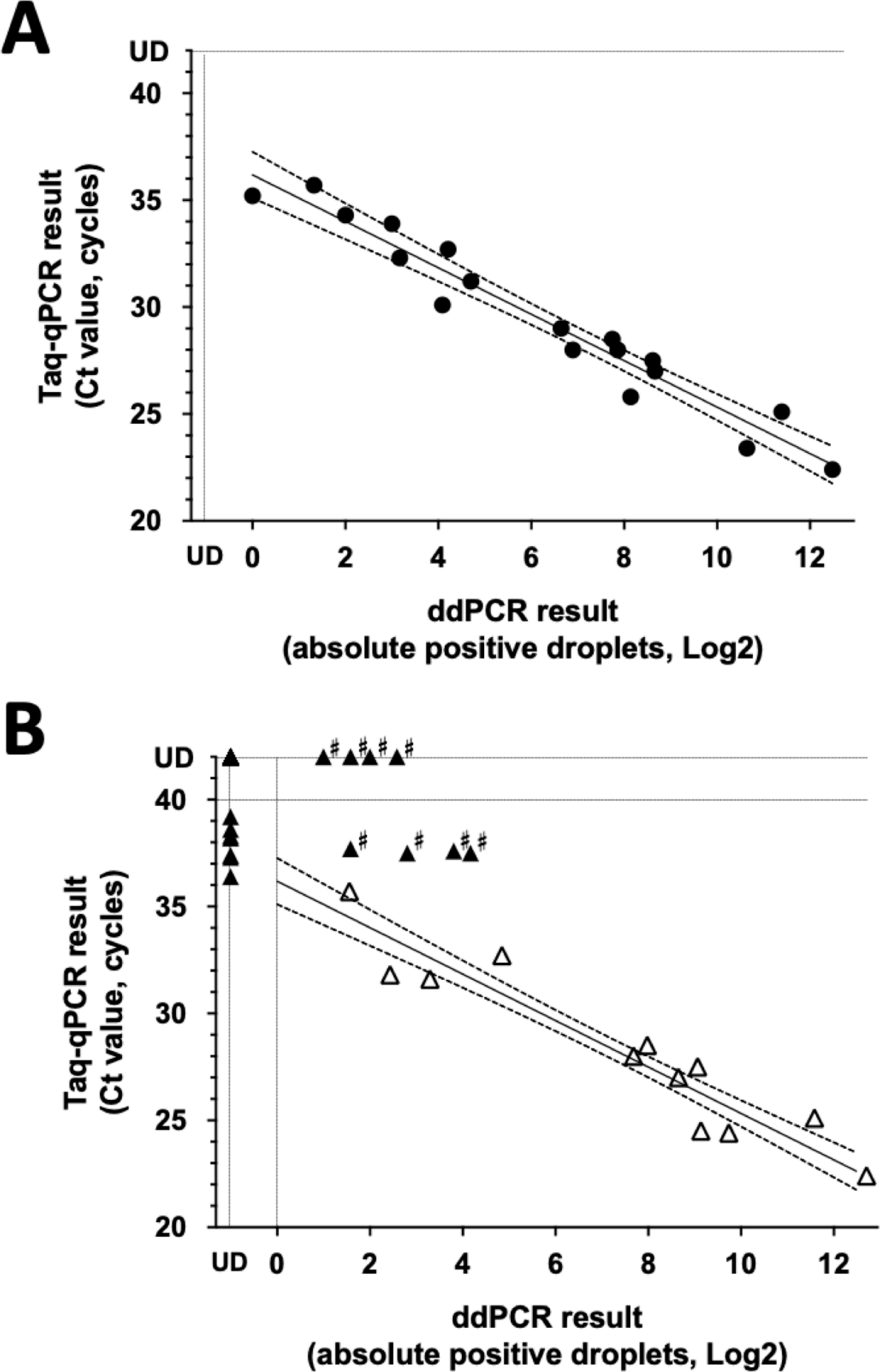
Primer-probe spefific Ct cut-off values for the laboratory strain of *E. histolytica* (A) and evaluation using clinical samples (B). A standard curve with 95% confidence intervals was drawn between Ct value by qPCR and square APD by ddPCR using a serial dilution of *E. histolytica* laboratory strain (R² = 0.9965, p < 0.0001). The primer-probe set 5 specific cut-off Ct value was determined as 36 cycles, which is the intersection of the standard curve on 1 APD by ddPCR (A). The APD and Ct value of clinical samples were directly plotted on the standard curve created in Fig 4A. Open triangles represent the results of the samples with a lower Ct than the cut-off Ct value (Ct ≤ 36 by qPCR). Filled triangles represent the results of the samples with a higher Ct than the cut-off Ct value (Ct > 36, or undetermined by qPCR). The samples plotted far outside the 95% CI are indicated by # (discordant PCR results), some of which were transferred to the shotgun metagenome analysis to check the existence of *Entamoeba* derived DNA (B).

Next, to determine cut-off Ct values specific to the primer-probe set, we applied ddPCR in combination with qPCR, which draws a standard curve between Ct value and APD. We performed ddPCR and qPCR using a series of different concentrations of *E. histolytica* HM-1 as templates and drew a standard curve between Ct value and APD (Fig 4A). As expected, Ct values were inversely proportion to the square of APD counts (R^2^ = 0.9965, p value < 0.0001) with 95% confidence intervals (CI). Finally, we determined the primer-probe specific cut-off Ct value as 36 cycles, which is the detection limit of one positive droplet count in ddPCR.

### Performance of qPCR cut-off Ct value for clinical specimens

To assess the diagnostic value of primer-probe set 5 and the accuracy of the cut-off Ct value, we performed ddPCR and qPCR for 66 clinical samples with various Ct values (S1 Data). We performed qPCR in duplicate and ddPCR four times for each sample to obtain reliable PCR results. Positivity was determined when positive results were obtained in all assays, and data was presented as the average titer.

For twelve samples with a lower Ct value than the cut-off Ct value (≤36), ddPCR was also positive. Moreover, APD was plotted around the 95%CI of the standard curve (represented as open triangles in Fig 4B). Next, we examined ddPCR using 11 template DNA samples with positive reactions and higher Ct than the cut-off by qPCR assay (36 < Ct < 40 cycles). Positive droplets were not observed by ddPCR in seven cases, indicating these samples represented false positives by qPCR. Surprisingly, in the other four cases, ddPCR identified an unexpectedly high number of APD. Also, we applied ddPCR for 43 qPCR negative cases. Interestingly, ddPCR identified additionally identified an unexpectedly high number of APD in four qPCR negative cases (named as discordant PCR cases indicated by # in Fig 4B).

Thus, ddPCR newly identified an unexpectedly high number of APD in some cases (discordant PCR cases). This indicated that fluorescence was generated in the PCR assay; however, fluorescence was not efficiently produced compared with that from the amplicon in the *E. histolytica* genome in these cases. In addition, to investigate the origin of the discordant PCR signals, we selected three representative cases (DDQ-10, DDQ-13, and DDQ-16) for shotgun metagenomic sequencing. These cases were selected based on sufficient remaining DNA volume, as well as their distinct patterns in qPCR/ddPCR results due to high APD in ddPCR despite negative or borderline qPCR results. Sequencing was conducted using the DNBSEQ platform, and analysis focused on identifying potential off-target amplification or contaminating organisms (Table 3). However, analyses could not be performed for all cases, because the sample volume was not sufficient. We confirmed that the reads derived from the positive control (DDQ-55) were matched to the *E. histolytica* X16142 gene. We identified many reads from DDQ-55 were scattered to 36 different contigs of the *E. histolytica* genome of the *E. histolytica* reference strain (Table 3 and S2 Data). However, reads derived from discordant PCR cases were never mapped to X16142 although some reads mapped to the *E. histolytica* whole genome. However, matched reads of these samples were mapped to the single contig. The sequence of the contig was the same as the other intestinal bacteria (99.8% similarity to *Clostridium perfringens* for NW_001915840, or 100% similarity to *Escherichia coli* for NW_001916323), indicating these reads were derived from these enteric bacteria, not from *Entamoeba spp*. Last, we examined sequences that might potentially match the forward or reverse primers. Many sequences from various intestinal organisms might match the primer sequences; however, the number of sequences was similar between the negative control and samples with discordant PCR cases. We could not identify the specific organism potentially amplified by the forward and reverse primer sets (Table 3 and S3 Data).

**Table 3:** Read sequence analysis of the template solutions.

	DDQ-55 (Proven *E. histolytica* positive control)	DDQ-47 (Proven *E. histolytica* negative control)	DDQ-13	DDQ-10	DDQ-16
Read information constructed from DNA library					
Raw reads by DNB-SEQ	26,352,760	38,636,884	38,606,267	39,019,871	24,169,710
Filtered after excluding reads with low quality	23,149,356	35,533,378	35,771,509	35,754,780	22,507,694
Mapping results to *E. histolytica* small subunit ribosomal RNA gene (X64142)*					
Number of mapped reads to X64142	4	0	0	0	0
BLAST search of mapped read sequence	100% matched to *E. histolytica* X64142	N/A	N/A	N/A	N/A
Mapping results to *E. histolytica* whole genome of HM1:IMSS*					
Number of mapped reads	67	0	13	2	25
Number of mapped contigs of *E. histolytica*	36	N/A	1***	1***	1****
Potentially amplifiable reads by primer-probe set 5					
Primer matching organisms** (forward/reverse primer)	39/41	101/194	44/31	54/15	41/8

*Conditions with length fraction (0.98) and similarity fraction (0.98) were applied. **Less than 2 nucleotide mismatches were permitted for matching conditions. ***All reads were mapped to the same contig (NW_001915840, total length 1771 bp), which had 99.8% similarity to *Clostridium perfringens*. ****All reads were mapped to the same contig (NW_001916323, total length 1031 bp), which had 100% similarity to *Escherichia coli* and other intestinal commensal bacteria.

Abbreviations: qPCR, quantitative PCR; UD, undetermined; BLAST, DNB-SEQ.

Taken together, combination use of ddPCR with qPCR revealed that the false positive reactions can commonly happen in stool specimens as discordant PCR results. Also, it was suggested that the qPCR assay with logically determined cut-off Ct can confer the accurate diagnosis of *E. histolytica* infection in the clinical settings. However, the source of the non-specific fluorescence reaction causing false positive reactions was not identified in the present analyses.

## Discussion

The main objective of this study was the optimization of qPCR for the clinical diagnosis of *E. histolytica* infection. Standard ddPCR protocol with 50 cycles showed that sufficient numbers of positive droplets were obtained from all primer-probe candidates, regardless of amplification efficiency. However, with lower PCR cycles, only a few primer-probe sets obtained the same number of APD as those obtained with 50 cycles, moreover, differences in MFI between efficient and inefficient primer-probe sets were clearer with reduced cycles. Finally, we determined the two most effective primer-probe sets from an initial pool of 20 candidates. Next, we identified a primer-probe specific cut-off Ct value by plotting a standard correlation curve between Ct value and square APD. The utility of selected primer-probe set (set 5) and its cut-off Ct value were subsequently validated with clinical samples, which showing that this approach is practical for the clinical diagnosis. Of note, our strategy can be widely applied to other pathogens in the clinical specimens. Moreover, prior information on the target DNA concentration in the template is not required when determining cut-off Ct values, suggesting cut-off Ct values could be determined even for uncultivable pathogens using clinical specimens of unknown concentrations.

An additional objective of this study was to assess the significance of qPCR positivity with higher Ct values above the cut-off value, as these are frequently encountered in clinical practice and are often challenging to interpret. We analyzed clinical specimens using ddPCR and qPCR. Generally, positive droplets were not identified by ddPCR in cases with a high Ct by qPCR, which indicated that qPCR produced false positive results. Surprisingly, some samples exhibited an unexpectedly high number of APD by ddPCR in samples with undetermined or high Ct values by qPCR (discordant PCR cases). We performed shotgun metagenome sequencing analyses, which confirmed that *Entamoeba-*related DNA was not detected in these samples. Thus, we demonstrated that the false positive reactions can be commonly observed in stool specimen, which could be identified by the concomitant use of ddPCR and qPCR for the same sample. However, we could not identify the cause of these reactions. One possibility is primer-probe was accidentally reacted by unexpected enzymatic activity induced by the factors in template solution from clinical specimens. We determined all PCR conditions (primer, probe, enzyme concentrations, etc.) by experiments using a reference strain (HM1:IMSS clone 6). However, it was impossible to optimize the PCR conditions for each clinical specimens, in which the presence of *E. histolytica* had never been known prior to the experiments.

This study had some limitations. First, the cause of false positive reactions generated by qPCR and/or ddPCR in stool specimens could not be verified. In the present study, PCR conditions, including primer-probe concentrations and annealing temperatures, were investigated using a reference strain (HM1:IMSS clone 6). Also, our protocol for clinical samples provides only a small amount of DNA template solution (50 μL) from each specimens. Therefore, we could not perform some verification experiments for identifying false positive reactions (e.g., PCR using concentrated template DNA, conventional (or nested) PCR followed by Sanger sequencing, and PCR targeting the other lesions of *Entamoeba* or different organisms). Instead, we performed shotgun metagenome analysis; however, we could not identify the exact cause of the discordant results. Research focusing on the specific samples of discordant PCR results will be expected to identify the cause of false positive reactions. Second, the sample composition in this study was predominantly stool samples with limited verification in other sample types. Future studies should extend the assay to include samples from other sources, such as liver abscesses and tissue biopsies, to confirm whether the calibration curve and cut-off values established here are applicable to a broader clinical context. In addition, future studies should validate this approach using clinical samples from developing countries, where co-infections with other *Entamoeba* species such as *E. dispar* or *E. moshkovskii* are more prevalent.

In conclusion, the combination use of ddPCR with qPCR revealed that false positive reactions of qPCR and/or ddPCR commonly happen in stool specimens. Also, this study emphasizes the value of ddPCR for establishing accurate cut-off values as well as assessing primer-probe efficiency for improving *E. histolytica* diagnosis in the clinical settings. Further studies should address non-specific fluorescent reactions in stools, and expand our strategy to a wider range of microbes and the other sample types for enhancing the diagnostic accuracy for infectious diseases.

## Supporting information

S1 Data Sample information of clinical specimens. (XLSX)

S2 Data Contig information of *E. histolytica* to which reads derived from clinical samples were mapped. (XLSX)

S3 Data Read sequences of other intestinal organisms that were potentially amplified by primer set 5. (XLSX)
